# Inhibition of RANKL-stimulated osteoclast differentiation by *Schisandra chinensis* through down-regulation of NFATc1 and c-fos expression

**DOI:** 10.1186/s12906-018-2331-5

**Published:** 2018-10-01

**Authors:** Eun-Jung Kim, Haesu Lee, Mi Hye Kim, Woong Mo Yang

**Affiliations:** 1 College of Korean Medicine, Dongguk University, Gyeongju, Republic of Korea; 2 Department of Convergence Korean Medical Science, College of Korean Medicine, Kyung Hee University, 26 Kyungheedae-ro, Dongdaemun-gu, Seoul, 02447 Republic of Korea

**Keywords:** *Schisandra chinensis*, Osteoclastogenesis, RANKL, NFATc1, C-fos

## Abstract

**Background:**

*Schisandra chinenesis* (SC) has been reported to have ameliorative effect on osteoporosis. However, the mechanisms underlying the anti-osteoporosis activity of SC have not been clearly elucidated. In the present study, we determined the effects of SC on The receptor activator of NF-kB ligand (RANKL)-induced osteoclastogenesis and its potential mechanism.

**Methods:**

Raw 264.7 cells were treated with 0.6, 6 and 60 μg/mL SC in the presence of 100 ng/mL RANKL for 7 days. RANKL-induced osteoclast formation was analyzed by tartrate resistant acid phosphatase (TRAP) staining. The osteoclast differentiation-related factors were confirmed along with TNF-α.

**Results:**

SC inhibits the RANKL-induced osteoclast differentiation in dose-dependent manner within non-toxic concentrations. The supernatant concentrations of TNF-α were significantly decreased by SC treatment. In addition, osteoclastogenesis-related factors, TRAP6 and NF-κB, were markedly decreased by SC in RANKL-induced osteoclasts. Mechanistically, SC reduced the RANKL-triggered NFATc1 and c-fos expressions.

**Conclusions:**

Taken together, our data suggest that SC can modulate bone metabolism by suppressing RANKL-induced osteoclast differentiation.

**Electronic supplementary material:**

The online version of this article (10.1186/s12906-018-2331-5) contains supplementary material, which is available to authorized users.

## Background

Osteoclast, the bone-resorbing multinucleated giant cells, developed from the monocyte-macrophage lineage cells [1]. Excessive osteoclast activity leads to an imbalance between bone resorption and formation, which is frequently observed in various osteopenic diseases such as osteoporosis [2], skeletal metastases [3], periodontitis [4], Paget’s disease [5] and rheumatoid arthritis [6].

The receptor activator of nuclear factor-kappa B ligand (RANKL) belongs to the tumor necrosis factor (TNF) receptor-ligand family, and is directly involved in the differentiation of osteoclasts through its receptor, RANK [7]. RANKL-induced stimulation of RANK on hematopoietic precursor cells leads to the recruitment of TNF receptor-associated factors (TRAF) and the following activation of several downstream signaling pathways such as nuclear factor-kappa B (NF-κB), mitogen-activated protein kinase (MAPKs), c-fos and nuclear factor of activated T cells (NFATc1), ultimately resulting in generating mature osteoclasts [8–11]. Therefore, the investigation of targeted modulation of RANKL signaling pathways to regulate the differentiation of osteoclasts may have significant therapeutic implications for the treatment of bone erosive diseases such as osteoporosis, periodontitis and osteoarthritis [12].

The fruit of *Schisandra chinensis* (Turcz.) Baill. (Schisandraceae) has been used for the treatment of rheumatoidal and degenerative arthritis in traditional Korean medicines [13]. *S. chinensis*, also known as Omija in Korea, has been widely harvested in East Asia including Korea, China, Japan and Russia [14]. *S. chinensis* contains several lignans, mainly schizandrin and gomisin A, which have been shown to possess a stimulating activity of osteoblastic proliferation in vitro [15, 16]. Recently, *S. chinensis* has been reported to exhibit an ameliorative effects against osteoporosis through the activation of estrogen receptors [17]. Several individual pathways including NF-κB and MAPKs pathways have been suggested for regulation of lipopolysaccharide-induced inflammation by treatment of *S. chinensis* or its lignans [18–20]. Interestingly, Schisantherin A, a main constituent of *Schisandra sphenanthera* Rehder & E. H. Wilson (Schisandraceae), not a predominant constituent of *S. chinensis*, is reported to suppress the osteoclast formation in vitro [21, 22]. However, the effects of *S. chinensis* on the differentiation of osteoclasts and their underlying mechanisms have not been fully clarified yet.

In the present study, we evaluated the effects of *S. chinensis* on RANKL-induced osteoclastogenesis and investigated the potential mechanisms of inhibiting osteoclasts differentiation by interfering with RANKL signaling pathways.

## Methods

### Preparation of SC

*S. chinensis* was purchased from OmizaValley Inc. (Mungyeong-si, Korea). The crude extract of *S. chinensis* was prepared by refluxing. 400 g of *S. chinensis* was boiled with distilled water at 100 °C for 2 h and filtered through 185 mm filter paper. The extract was lyophilized and called SC. The final yield was 35%. A voucher specimen (SC-W100) of plant was deposited in the college of Korean Medicine, Kyung Hee University, Seoul, Korea. The quality evaluation of SC was determined by high-performance liquid chromatography with evaporative light scattering detector (HPLC-ELSD; HPLC Agilent 1100 series). Shizandrin was used as a marker of SC. Thirty mg SC was dissolved in 1 mL 30% ethanol and sonicated for 30 min. Following filtering through a 0.45 μm filter membrane, 10 μL of 2 fold diluted aliquot was injected to HPLC-ELSD system equipped with a Atlantis HILIC silica (4.6 × 150 mm, 5 μm, 100 Å). The correlation coefficient (R2) reached 0.9999. The concentration of schizandrin in SC was 164.372 μg/mL (1.820%) shown in Additional file 1.

### Osteoclast formation

The murine RAW 264.7 cells were seeded at a density of 8 × 10^4^ cells per well in 6 well plates. The growing medium is α-Minimum Essential Medium Eagle (α-MEM) supplemented with 10% heat inactivated fetal bovine serum (FBS) and 100 units/mL penicillin. To generate osteoclasts, all cells without non-treated cells were incubated with RANKL. Then RANKL-induced cells were either left untreated or treated with various concentrations of SC 0.6, 6 or 60 μg/mL for 7 days. Non-treated cells were not treated RANKL and SC as normal control. The media was replaced after 3 days. Tartrate resistant acid phosphatase (TRAP) staining was performed in 7 days using a commercial kit (Sigma, MO, USA). Cells were treated with TRAP staining solution including 1% naphthol AS-BI phosphate, 2% diazotized Fast Garnet GBC solution in sodium nitrite, 4% acetate solution and 2% tartrate solution for 30 min. TRAP-stained cells were counterstained by hematoxylin and monitored under a light microscope using the Leica Application Suite (LAS; Leica Microsystems, Buffalo Grove, IL, USA). Osteoclasts were defined as TRAP-positive multinucleated cells (> 3 nuclei/cell). To quantify the TRAP intensity, each well was added 400 μL of citrate solution including sodium tartrate and p-nitrophenylphosphate. After 1 h, supernatant was collected, 400 μL of 0.1 N sodium hydroxide was added and measured at 410 nm using a microplate reading instrument. The experiments were carried out 3 times in triplicate measurements. The cytotoxicity of Raw 264.7 cells was confirmed by 3-(4,5-Dimethylthiazol-2-yl)-2,5-Diphenyltetrazolium Bromide (MTT) assay. Raw 264.7 cells were treated in the presence of 0.6, 6 and 60 μg/mL SC for 24 h. 2 mg/mL MTT solution was added into cells and color density at 570 nm absorbance was detected by a microplate reading instrument.

### Enzyme-linked immunosorbent assay (ELISA)

The supernatants derived from osteoclasts were collected at 7 days after RANKL and SC treatment. The concentration of TNF-α was quantified using TNF-α ELISA kit (BD Bioscience, San Jose, CA, USA) according to the manufacturer’s protocol. Color development at 450 nm was measured.

### Western blotting analysis

RANKL-induced osteoclast was prepared as mentioned above. On the 7 days after osteoclast induction, cells were lysed with RIPA buffer (Pierce Biotechnology, Rockford, IL, USA) containing protease inhibitors (Roche, Hoffmann, USA). 30 μg protein samples were separated in sodium dodecyl sulfate-polyacrylamide gel, and transferred to polyvinylidene fluoride membrane (Bio-Rad, Hercules, CA, USA). Each membrane was incubated with primary β-actin, TRAF6, NF-κB, Lamin B, IκB-α, p-IκB-α, extracellular signal–regulated kinase (ERK), c-Jun N-terminal kinase (JNK), p38, NFATc1 and c-fos antibodies (Cell Signaling, USA) overnight at 4 °C. Anti-mouse IgG was used as the secondary antibody. Immunoreactivity was detected using an enhanced chemiluminescence detection system. The experiments were carried out 3 times in triplicate measurements.

### Statistical analysis

Significance was determined by one-way analysis of variance (ANOVA) and Dunnett’s multiple comparison tests. In all analyses, *P < 0.05* was taken to indicate statistical significance.

## Results

### SC suppressed RANKL-induced osteoclast formation

**Fig. 1:**
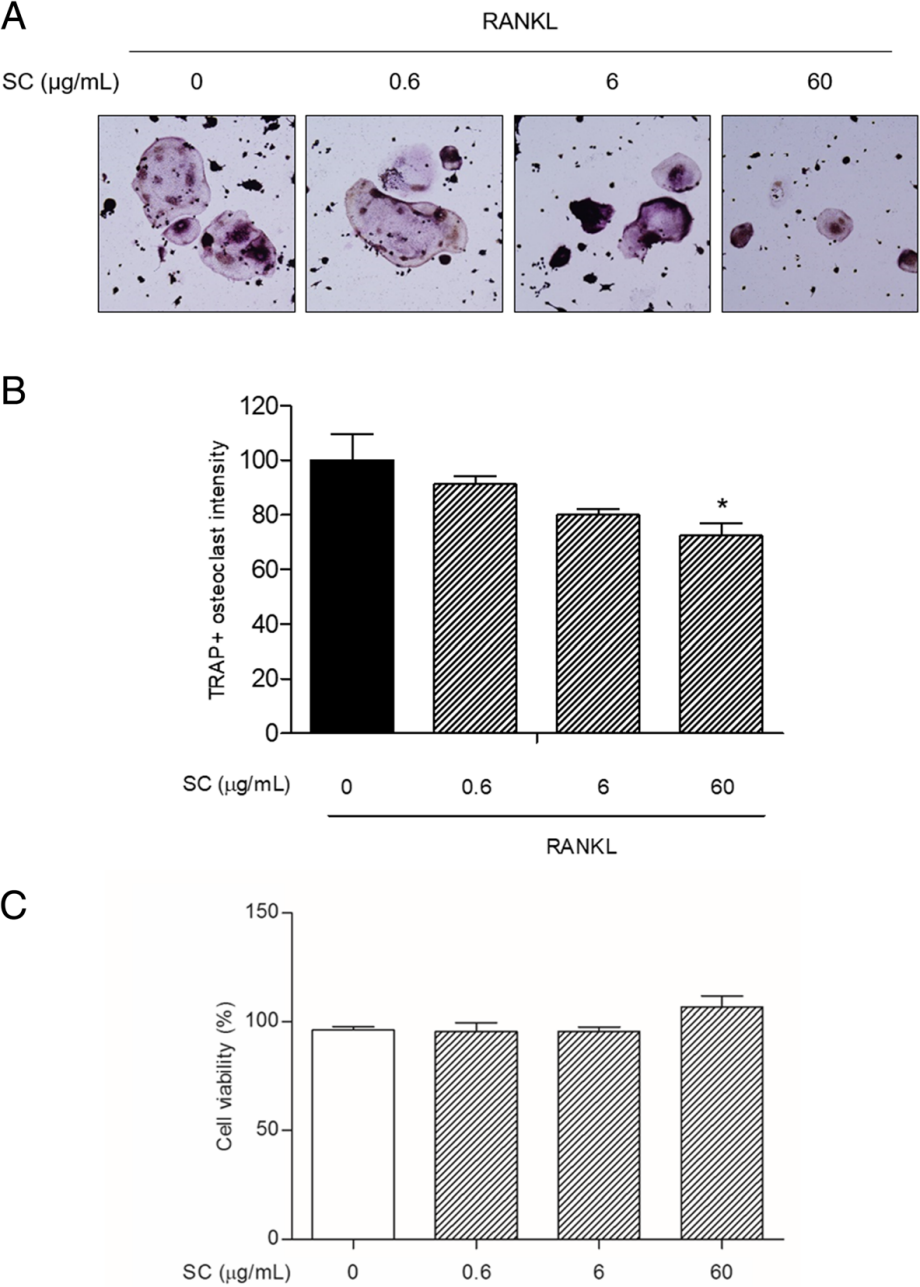
The effects of SC on osteoclast differentiation in RANKL-stimulated RAW 264.7 cells. **a** RAW 264.7 cells (8 × 10^4^ per well) were incubated with 100 ng/mL RANKL or both RANKL and SC (0.6, 6 and 60 μg/mL) for 7 days and then stained for TRAP. Magnification, 100× original. **b** TRAP activity was measured using an ELISA reader (optical density, 410 nm). Data are represented as the means ± S.E.M. of three independent experiments. ^*^ *p* < 0.05 compared with only RANKL-treated cells. **c** Cell viability was confirmed by MTT assay

Numerous mature multinucleated TRAP-positive osteoclasts were seen in RANKL-stimulated cells. SC treatment significantly decreased osteoclast differentiation as shown in images by light microscope (Fig. 1a). Also, SC treatment showed the inhibition of RANKL-induced osteoclastogenesis in a dose-dependent manner (17.06, 20.76 and 30.77%, respectively), as determined by measuring the cellular TRAP activity. All concentrations of SC exerted equivalent effects on cell viability. 0.6, 6 and 60 μg/mL SC treatment had no cytotoxicity to RAW 264.7 cell (Fig. 1b).

### SC suppressed RANKL-induced TNF-α secretion

**Fig. 2:**
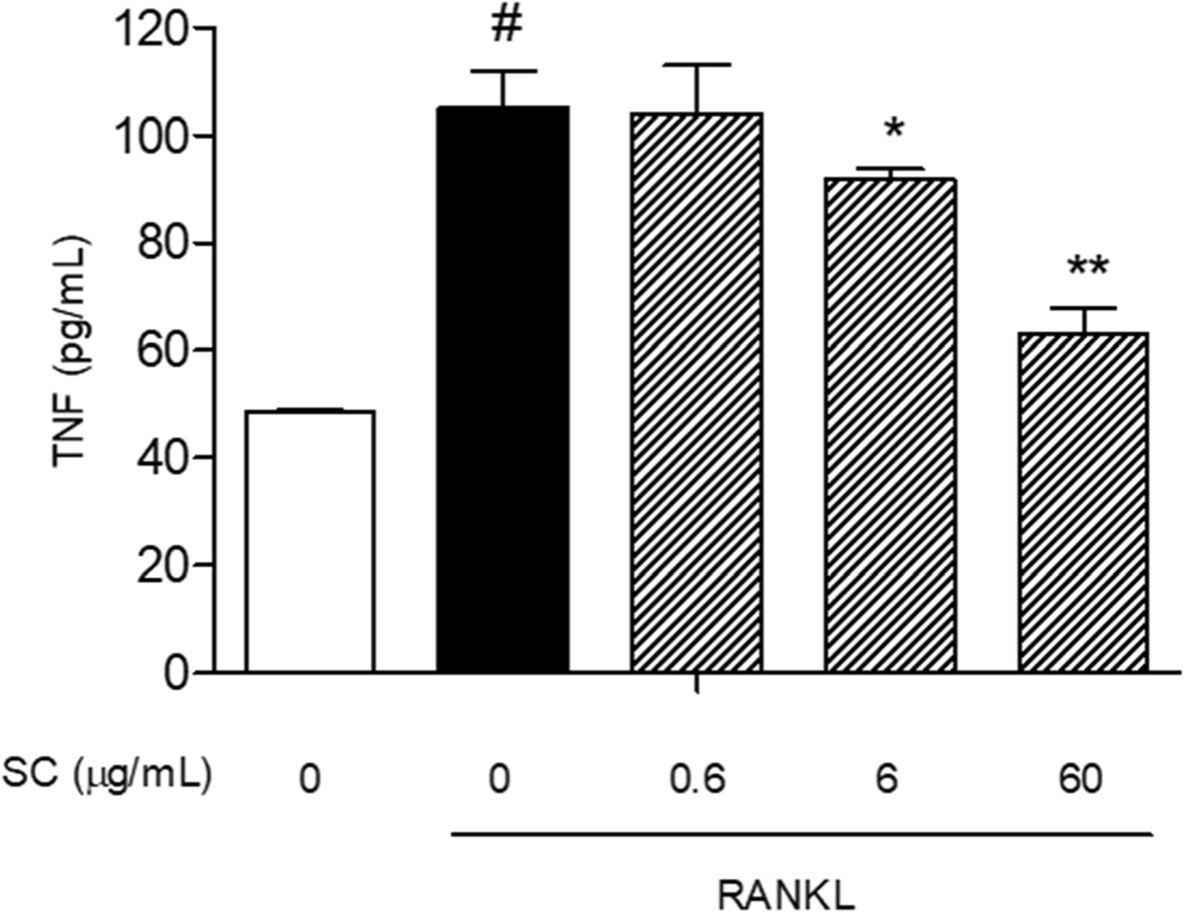
The effect of SC on TNF-α secretion in RANKL-stimulated RAW 264.7 cells. RAW 264.7 cells (8 × 10^4^ per well) were incubated with 100 ng/mL RANKL or both RANKL and SC (0.6, 6 and 60 μg/mL). After 7 days, the supernatant was collected and centrifuged. Total TNF-α concentration was calculated by ELISA. Data are represented as the means ± S.E.M. of three independent experiments. ^#^ *p* < 0.05 compared with non-treated cells. ^*^ *p* < 0.05 and ^**^ *p* < 0.01 compared with only RANKL-treated cells

RANKL increased the concentration of TNF-α in Raw 264.7 cells. Cells treated with SC decreased the secretion of TNF-α compared with cells treated with RANKL alone (Fig. 2). Especially, the levels of TNF-α were significantly reduced at the concentrations of 6 and 60 μg/mL SC. The inhibition rates of TNF-α secretion by 6 and 60 μg/mL SC were 19.3 and 27.6%, respectively.

### SC suppressed RANKL-induced TRAF6 expression

**Fig. 3:**
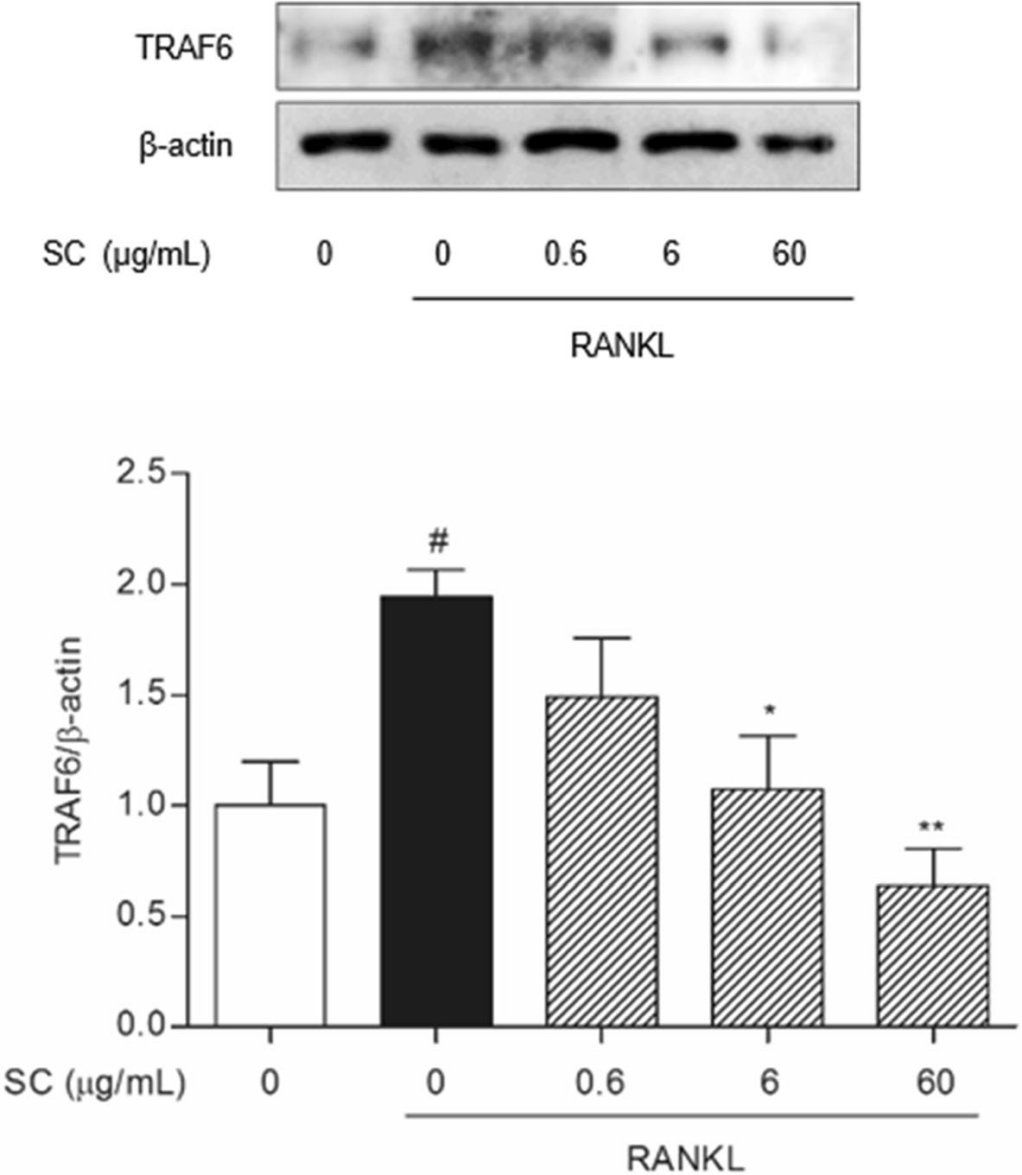
The effect of SC on TRAF6 protein expression in RANKL-stimulated RAW 264.7 cells. RAW 264.7 cells (8 × 10^4^ per well) were incubated with 100 ng/mL RANKL or both RANKL and SC (0.6, 6 and 60 μg/mL). After 7 days, total protein was then isolated and protein expression levels were evaluated by Western blot assay. Data are represented as the means ± S.E.M. of three independent experiments. ^#^ *p* < 0.05 compared with non-treated cells. ^*^ *p* < 0.05 and ^**^ *p* < 0.01 compared with only RANKL-treated cells

Since TRAF6 is directly recruited by RANK-RANKL complex and related with TNF-α signaling, we confirmed the protein expression of TRAF6 in RANKL-induced Raw 264.7 cells. The protein level of TRAF6 was significantly increased by RANKL treatment. Otherwise, SC at 6 and 60 μg/mL concentrations exhibited a marked inhibitory effect on TRAF6 expression in RANKL-induced Raw 264.7 cells (Fig. 3).

### SC suppressed RANKL-induced NF-κB translocation and IκB-α phosphorylation, not MAPKs phosphorylation

**Fig. 4:**
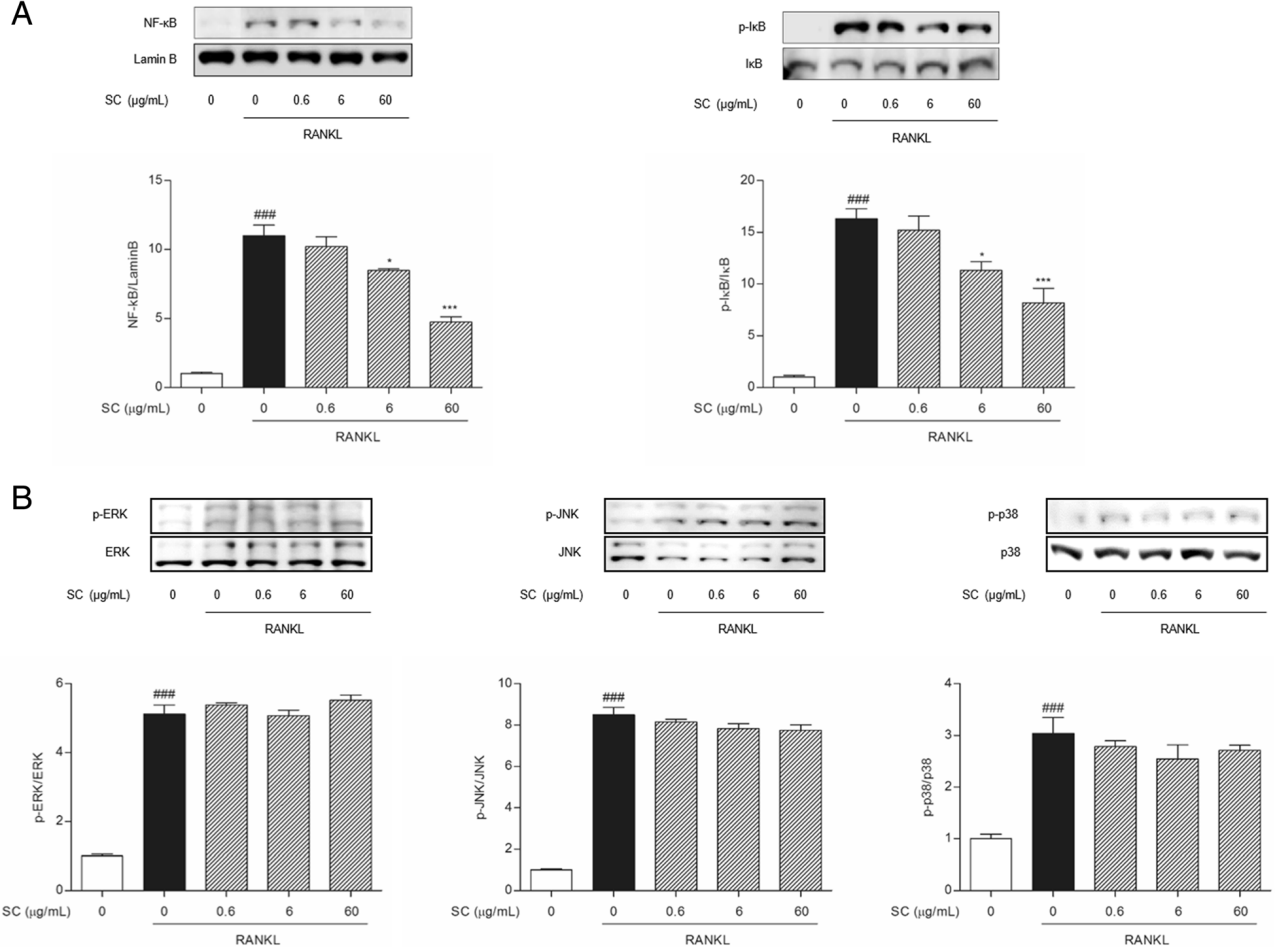
The effects of SC on (**a**) NF-κB and IκB-α, and (**b**) MAPKs pathway-related protein expressions in RANKL-stimulated RAW 264.7 cells. RAW 264.7 cells were cultured in the presence of RANKL with the vehicle or SC. After 7 days, total protein was then isolated and protein expression levels were evaluated by Western blot assay. Data are represented as the means ± S.E.M. of three independent experiments. ^###^ *p* < 0.001 compared with non-treated cells. ^*^ *p* < 0.05 and ^***^ *p* < 0.001 compared with only RANKL-treated cells

RANKL stimulation induced the translocation of NF-κB into nucleus and the phosphorylation of IκB-α in cytoplasm. The expressions of NF-κB in nuclear protein were reduced by treatment with 6 and 60 μg/mL SC (Fig. 4a). Similarly, SC co-treated with RANKL inhibited the phosphorylation of IκB-α in cytoplasmic protein compared to cells treated with RANKL alone. In addition, we analyzed the expressions of MAPKs such as ERK, JNK and p38 in RANKL-induced osteoclasts. Compared to non-treated cells, RANKL treatment showed increments of ERK, JNK and p38, respectively. However, SC did not alter RANKL-induced MAPKs expression (Fig. 4b).

### SC suppressed RANKL-induced osteoclast-specific transcription factors

**Fig. 5:**
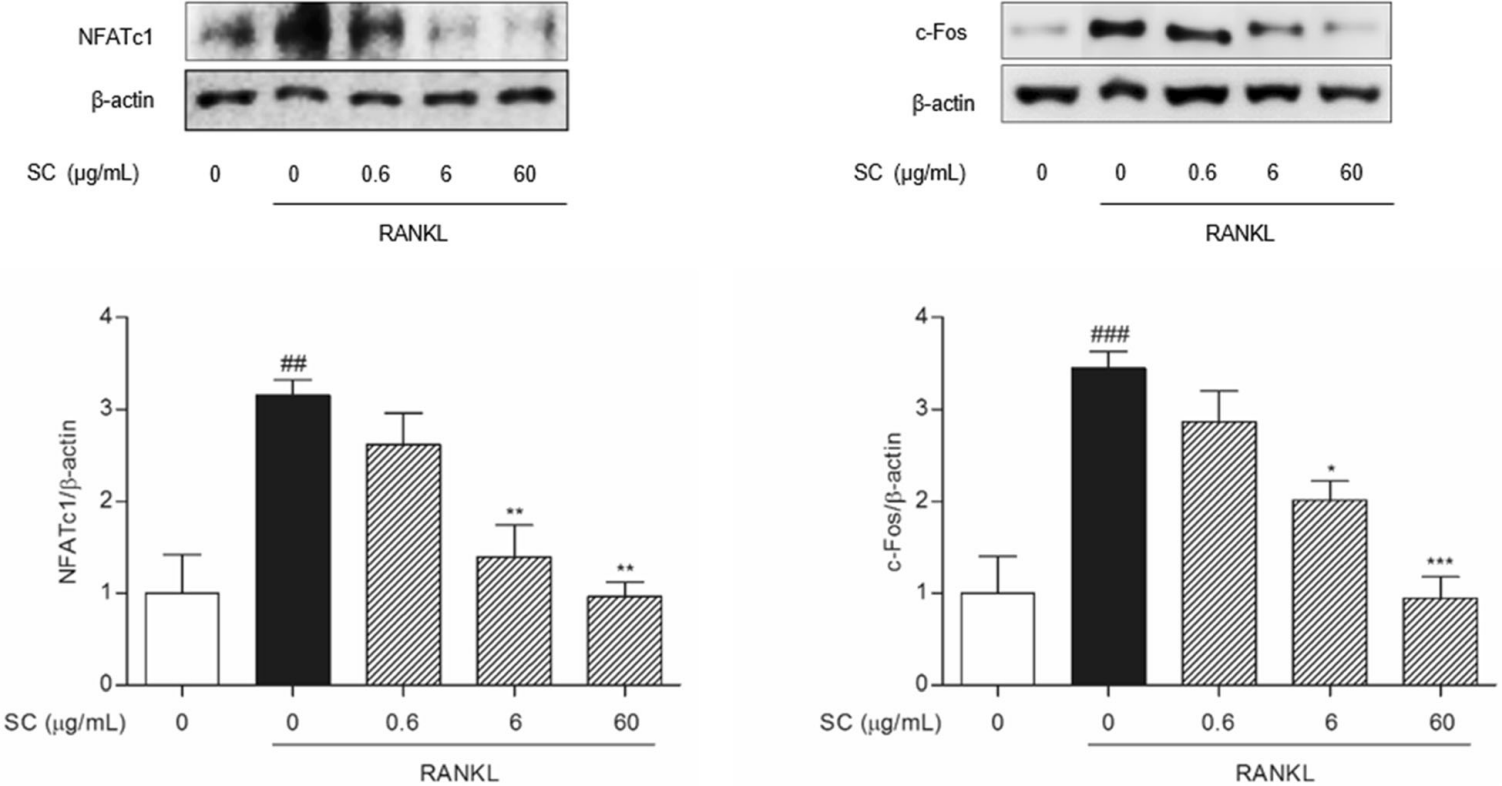
The effects of SC on NFATc1 and c-fos protein expressions in RANKL-stimulated RAW 264.7 cells. RAW 264.7 cells were cultured in the presence of RANKL with the vehicle or SC. After 7 days, total protein was then isolated and protein expression levels were evaluated by Western blot assay. Data are represented as the means ± S.E.M. of three independent experiments. ^##^ *p* < 0.01 and ^###^ *p* < 0.001 compared with non-treated cells. ^*^ *p* < 0.05, ^**^ *p* < 0.01 and ^***^ *p* < 0.001 compared with only RANKL-treated cells

To further define the mechanisms underlying the inhibitory effects of SC on NF-κB activation, the effects of SC on RANKL-induced osteoclast-specific transcription factors such as NFATc1 and c-fos were investigated. As shown in Fig. 5, the expression of NFATc1 was dose-dependently down-regulated in SC-treated cells. In addition, increased c-fos expressions in RANKL-induced osteoclast were significantly suppressed by treatment with 6 and 60 μg/mL SC.

## Discussion

The bone loss is caused by enhanced bone resorption with excessive RANKL signaling [23]. As such, inhibiting the formation of bone-resorbing osteoclasts through suppressing the RANKL signaling or its downstream pathways should be a rational target for the treatment of osteopenic diseases. In the present study, TRAP activities were measured in RANKL-treated Raw 264.7 cells to evaluate the effects of SC on osteoclast differentiation. RANKL induced the development of multinucleated osteoclasts from precursors, and SC treatments exerted preventive effects on the formation of the TRAP-positive osteoclasts without cytotoxicity.

TNF-α mediates RANKL stimulation of osteoclast differentiation by an autocrine mechanism [24]. Recently, it has been reported that TNF-α directly induces the osteoclastogenesis and bone loss. Since both RANKL and TNF-α can activate the same downstream signaling pathways such as NF-κB signal and MAPKs cascades [25], the effect of TNF-α on osteoclasts activation is strongly synergistic with RANKL. Our results showed SC (6 and 60 μg/mL) inhibited RANKL-induced TNFα secretion in RAW 264.7 cells. These results indicate that SC acts directly on osteoclast precursors to suppress osteoclast formation.

TRAF6-mediated signals play a key role in RANKL-induced signaling and osteoclast differentiation [26]. Binding of RANKL to RANK recruits TRAF6, forms an intermediate complex, which, in turn, can regulate NF-κB and MAPKs signaling pathways [27]. TRAF6 is presently known to be involved also in TNF-α signaling [28]. In the present study, enhanced TRAF6 expression in RANKL-induced osteoclasts was deceased by SC treatments (6 and 60 μg/mL), indicating that SC inhibits osteoclast differentiation via down-regulating the activity of TRAF6-dependent mechanism.

NF-κB pathway is one of the major intercellular pathways of osteoclasogenesis from precursors directly induced by RANKL-RANK-TRAF6 signaling axis [29]. It is well established that NF-κB pathway in RANKL-induced activation is essential for NFATc1 and c-fos expressions and early osteoclast differentiation [30, 31]. In the present study, SC inhibited the phosphorylation of IκB-α and the expressions of NF-κB in RANKL-induced osteoclasts (6 and 60 μg/mL), which means that SC could inhibit RANKL-induced activation of NF-κB pathway.

Besides the NF-κB pathway, MAPKs pathways, including ERK, JNK and p38, can be also stimulated by RANKL signaling [27]. Specific inhibitors of MAPKs pathways exhibit the effects of profound suppressions on RANKL-induced osteoclastogenesis from precursor cells [32–34]. Intriguingly, we observe no evidence that SC could inhibit RANKL-induced MAPKs pathway during osteoclastogenesis. Our results showed ERK, JNK and p38 phosphorylation was induced by RANKL stimulation, and SC treatments did not alter the increased phosphorylation of RANKL-induced MAPKs. These results seem to indicate that SC treatments inhibited RANKL signaling independently from MAPKs pathways.

NFATc1 and c-fos are specific and indispensable transcription factors for osteoclast formation [35, 36]. These factors are regarded two of the most important transcription factors in osteoclastogenesis, and lack of any of these two components blocks osteoclast formation. NFATc1, as a master regulator downstream of c-fos, NF-κB and MAPKs, integrates RANKL signaling in terminal differentiation of osteoclasts [37]. NFATc1 cooperates with c-fos to activate the autoamplification of NFATc1 itself and the transcription of osteoclast-specific genes including calcitonin receptor, TRAP, matrix metallopeptidase 9, and cathepsin K [38–40]. Expression of c-fos is modulated by various signaling pathways such as NF-κB, MAPKs, phosphatidylinositol 3-kinase-Akt and calcium/calmodulin-dependent kinase IV-cAMP response element-binding protein pathways [41]. In this study, SC inhibited the expressions of NFATc1 and c-fos in RANKL-induced osteoclasts. These results suggest that SC could suppress the osteoclastogenesis through osteoclast-specific transcriptional regulation.

## Conclusions

In conclusion, our current data demonstrate that SC attenuates RANKL-induced RAW 264.7 cells differentiation into osteoclasts. Furthermore, the effects of SC were associated with the inhibition of TRAF6 recruitment and inactivation of NF-κB pathway, but not MAPKs pathways, leading to the down-regulations of transcription factors including c-fos and NFATc1. This suggests that *S. chinensis* could be a promising therapeutic agent for osteopenic disorders.

## Additional file

Additional file 1: HPLC chromatograms of SC. The concentration of schizandrin in SC was 164.372 μg/mL (1.820%). (TIF 249 kb)

## Abbreviations

ERK: Extracellular signal–regulated kinase
JNK: C-Jun N-terminal kinase
MAPKs: Mitogen-activated protein kinase
NFATc1: Nuclear factor of activated T cells
NF-κB: Nuclear factor-kappa B
RANKL: Receptor activator of NF-kB ligand
SC: Water extract of *Schizandra chinensis*
TNF: Tumor necrosis factor
TRAF: TNF receptor-associated factors
TRAP: Tartrate resistant acid phosphatase
