# Supplementary figures and images for: Inhibition of RANKL-stimulated osteoclast differentiation by Schisandra chinensis through down-regulation of NFATc1 and c-fos expression
Source: BMC Complement Altern Med. 2018 Oct 1;18:270. doi: 10.1186/s12906-018-2331-5 (PMC6167898; doi:10.1186/s12906-018-2331-5)

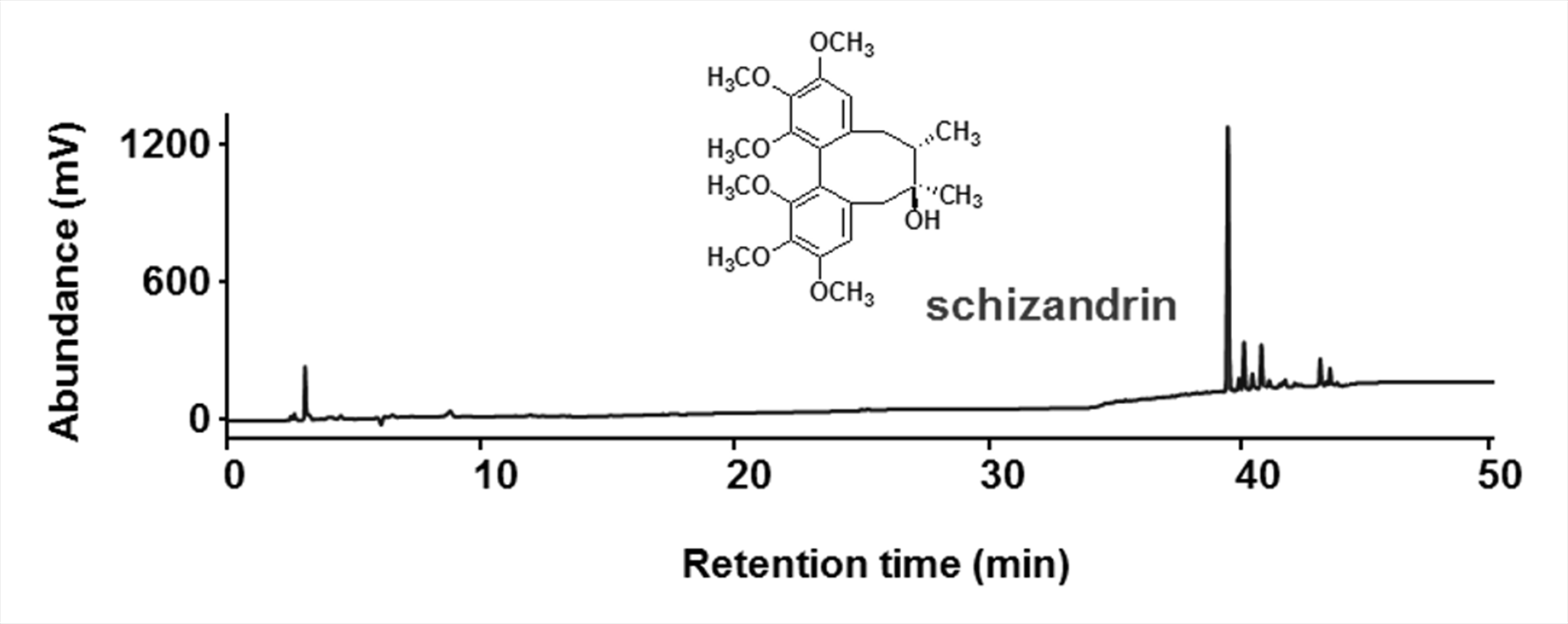

Supplement: Supplementary file 1 — HPLC chromatograms of SC. The concentration of schizandrin in SC was 164.372 μg/mL (1.820%). (TIF 249 kb) [file 12906_2018_2331_MOESM1_ESM.tif]
